# Developing a national atlas to support the progressive control of tsetse-transmitted animal trypanosomosis in Kenya

**DOI:** 10.1186/s13071-020-04156-5

**Published:** 2020-06-05

**Authors:** Nancy N. Ngari, Daniel O. Gamba, Pamela A. Olet, Weining Zhao, Massimo Paone, Giuliano Cecchi

**Affiliations:** 1 Kenya Tsetse and Trypanosomosis Eradication Council (KENTTEC), Nairobi, Kenya; 2 Food and Agriculture Organization of the United Nations (FAO), Animal Production and Health Division, Rome, Italy

**Keywords:** Tsetse, *Glossina*, African animal trypanosomosis, Kenya, Epidemiology, Animal health, Progressive control pathway, Database, Atlas, GIS

## Abstract

**Background:**

African animal trypanosomosis (AAT) is a major livestock disease in Kenya. Even though, over the years various organizations have collected a vast amount of field data on tsetse and AAT in different parts of the country, recent national-level maps are lacking. To address this gap, a national atlas of tsetse and AAT distribution is being developed by the Kenya Tsetse and Trypanosomosis Eradication Council (KENTTEC) and partners.

**Methods:**

All data collected by KENTTEC from 2006 to 2019 were systematically assembled, georeferenced and harmonized. A comprehensive data repository and a spatially-explicit database were created. Input data were collected mainly in the context of control activities, and include both baseline surveys (i.e. pre-intervention) and the subsequent monitoring during and after interventions. Surveys were carried out in four regions (i.e. Western, Rift Valley, Central and Coast), and in 21 of the 47 counties in Kenya. Various devices were used for entomological data collection (i.e. biconical, NGU and H traps, and sticky panels), while the buffy-coat technique was the method used to detect AAT.

**Results:**

Tsetse trapping was carried out in approximately 5000 locations, and flies (> 71,000) were caught in all four investigated regions. Six species of *Glossina* were detected: *G. pallidipes* (87% of the catches); *G. brevipalpis* (8%); *G. fuscipes fuscipes* (4%); *G. longipennis* (< 1%); *G. austeni* (< 1%); and *G. swynnertoni* (< 1%). A total of 49,785 animals (98% of which cattle) were tested for AAT in approximately 500 locations. Of these, 914 animals were found to be infected. AAT was confirmed in all study regions, in particular caused by *Trypanosoma vivax* (48% of infections) and *T. congolense* (42%). Fewer cases of *T. brucei* were found.

**Conclusions:**

The development and regular update of a comprehensive national database of tsetse and AAT is crucial to guide decision making for the progressive control of the disease. This first version of the atlas based on KENTTEC data has achieved a remarkable level of geographical coverage, but temporal and spatial gaps still exist. Other stakeholders at the national and international level will contribute to the initiative, thus improving the completeness of the atlas. 
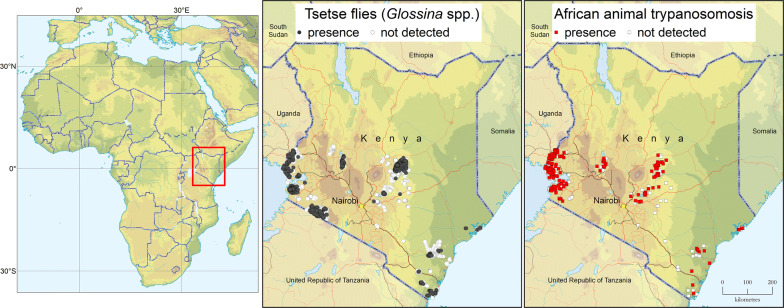

## Background

Tsetse flies (genus *Glossina*) are blood sucking insects responsible for the cyclical transmission of African animal trypanosomosis (AAT) [1] and human African trypanosomosis (HAT) [2]. AAT, also known as nagana, is a major constraint to agricultural production in Africa, with over 50 million cattle exposed to the risk of infection [3], and severe impacts in terms of underutilization of arable land and pastures [4]. The direct effects of the disease include increased livestock mortality, reduced milk yield, low live weight gain, abortions, infertility, and increased susceptibility to other diseases [5]. It has been estimated that the presence of AAT reduces the total number of livestock in an area by between 25 and 50% [6].

In Kenya, tsetse flies are estimated to affect 38 out of 47 counties [7], infesting an area of approximately 136,000 km^2^ (i.e. less than a quarter of the total country area) [8]. However, a much larger area in the country is at risk of AAT because of the possibility of mechanical transmission by other biting flies (e.g. tabanids and *Stomoxys*) and the movement of infected animals [9]. Overall, Kenya is estimated to lose over USD 200 million annually because of AAT [7]. Furthermore, by primarily affecting poor communities, the disease impacts already impoverished livestock farmers and threatens food security and livelihoods. Finally, tsetse infestation and trypanosomosis infection also have negative impacts on wildlife health and tourism.

The human form of trypanosomosis, HAT (also known as sleeping sickness) is a neglected tropical disease that threatens approximately sixty million people in Africa [10, 11]. In Kenya, the zoonotic rhodesiense form is present, but disease risk is generally considered low [10, 12, 13]. Autochthonous cases used to be reported from the western part of the country, in particular in transboundary foci with Uganda [14–16]. However, in the past ten years, only two HAT cases were reported in Kenya, both in tourists who had visited the Maasai Mara National Reserve [13].

Eight species of tsetse fly are historically reported to be present in Kenya: *G. brevipalpis*; *G. longipennis* and *G. fuscipleuris* (fusca/forest group, subgenus *Austenina*); *G. pallidipes*; *G. austeni*; *G. swynnertoni* and *G. morsitans submorsitans* (morsitans/savannah group, subgenus *Glossina* (*sensu stricto*)); and *G. fuscipes fuscipes* (palpalis/riverine group, subgenus *Nemorhina*) [17, 18]. With the exception of *G. fuscipleuris* and *G. morsitans submorsitans*, the presence of these species has been confirmed in a review of the scientific literature that covered the period 1990–2014 [19], and a more recent study also confirmed the presence of *G. fuscipleuris* [20]. Due to a combination of topographical, climatic, environmental and anthropogenic factors, the distribution of tsetse flies in Kenya is known to be fairly fragmented [21–23]

In regard to AAT, the major trypanosome species affecting livestock in Kenya are *Trypanosoma congolense*, *T. vivax*, *T. brucei* and *T. simiae*. Mechanical vectors are known to contribute to the transmission of trypanosomes, especially *T. vivax* [24, 25].

Over many years, various approaches and tools have been applied in different parts of the country in an effort to control tsetse and trypanosomosis. These include: bush clearing [26]; erection of tsetse barriers; ground spraying; aerial spraying [27]; odour baited insecticide-treated targets and traps (ITT); and live bait techniques (i.e. insecticide-treated cattle, ITC). The activities were often successful but not sustainable, leading to a resurgence of the problem when control activities were interrupted [27].

In the year 2000, the African Heads of State and Government passed a resolution aiming to eradicate tsetse and trypanosomosis from Africa. This triggered the launch of the Pan-African Tsetse and Trypanosomosis Eradication Campaign (PATTEC), coordinated by the African Union [28]. Kenya embarked on the initiative, and in 2005 it established the PATTEC Unit under the Department of Veterinary Services. The PATTEC project spearheaded efforts to eliminate tsetse and trypanosomosis in Kenya until 2011. In this six-year period, field activities focused on three tsetse-infested regions (i.e. Western, Rift Valley and Central). These are some of the areas where the AAT burden, and the benefits of control, are estimated to be highest [8]. In 2012, the Kenya Tsetse and Trypanosomosis Eradication Council (KENTTEC) was established to safeguard the gains made under the PATTEC project, to expand interventions to other priority areas (e.g. the Coast region), and to coordinate tsetse and trypanosomosis control at the national level.

Evidence-based planning and execution of control activities require reliable maps of tsetse and trypanosomosis distribution [29]. However, adequate information at the continental level is only available for HAT [13, 16], whilst there is a dearth of harmonized, large-scale georeferenced information on tsetse and AAT. The latest tsetse distribution maps at the continental level were developed in 1970s [30]. Subsequent continental mapping exercises focused on geospatial modelling rather than the assembly of more recent field data [21, 31]. With regard to AAT, Africa-level maps have never been developed. To address this gap in Africa-wide tsetse and AAT maps, FAO recently embarked on the development of a continental atlas using data from scientific publications [19, 32, 33]. The continental atlas of tsetse and AAT has not been completed yet, even though maps for selected countries, including Kenya, have been released (www.fao.org/paat).

In Kenya, the latest national map of tsetse distribution was generated more than twenty years ago [23], and AAT maps are lacking. In response to this issue, in 2016 KENTTEC launched the development of a national atlas of tsetse and AAT. The initiative is supported by FAO in the framework of the Programme Against African Trypanosomosis (PAAT) [34, 35]. The adoption in Kenya of the Progressive Control Pathway (PCP) for AAT [36] provided further thrust to the development of the atlas. PCPs are staged approaches to plan and evaluate progress in the reduction, elimination and eradication of a range of diseases [37, 38]. The establishment of a national-level, spatially explicit information system on tsetse and AAT (i.e. an atlas) is one of the key requirements to advance along the PCP. In particular, a comprehensive, harmonized database is needed to assess the PCP status both at national and sub-national level, and to inform decision making for disease control.

## Methods

The methodology used in developing the national atlas of tsetse and AAT for Kenya is broadly based on the FAO continental atlas [19, 32, 33]. The main difference being that the continental atlas solely relies on peer-reviewed scientific publications, whilst a national atlas should include all data collected in the country, be the data published or unpublished [9, 39].

### Input data

At the present stage of development, the atlas for Kenya is based on a large dataset of unpublished data collected by the PATTEC project/KENTTEC in the context of surveillance and control activities. All data included so far were collected between 2006 and 2019. Inputs include both baseline data (pre-intervention) and monitoring data (both during and post-intervention). Control activities and the related surveys focused on four intervention regions and 21 counties, i.e.: Western region (in particular Bungoma, Busia, Siaya, Kisumu, Homa Bay and Migori counties); Rift Valley region (Baringo and Narok counties); Central region (Isiolo, Meru, Tharaka-Nithi, Embu, Muranga, Machakos, Kitui and Makueni counties); and Coast region (Lamu, Taita-Taveta, Tana River, Kilifi and Kwale counties).

#### Tsetse data

Baseline data on tsetse flies were collected before the start of control interventions, with a view to ascertaining tsetse presence, abundance and distribution. Monitoring data were collected during (or after) the application of the different control techniques, to assess the effectiveness and the impact of interventions. The main tsetse control tools used during the study period are ITC, ITT [40–42] and livestock protective fences [43], which are considered to be particularly cost-effective for AAT control [44].

Different devices were used to trap tsetse flies in the different intervention areas, based on the locally prevailing tsetse species. These included biconical traps [45], NGU traps [46], H traps [47] and sticky panels [48]. Traps were baited with phenol (1:4:8) and acetone, and deployed in suitable tsetse habitats. In some areas, traps were deployed in the same locations at different periods (repeated monitoring), with a frequency ranging from monthly, quarterly, annually to biennially. In other areas, spot-checks were conducted (one-time monitoring). Both for repeated and one-time monitoring, traps were normally maintained in the position for 48 or 96 h before flies were harvested.

Field data were recorded in standard data sheets [49]. The sheets capture information such as the name of the surveyed area, administrative units, coordinates of the specific trapping site (as measured with GPS), date of survey, time and duration of trap deployment and harvesting. Finally, the number, age, and sex of the trapped tsetse flies is recorded by species. A data sheet normally includes results for 10 to 20 traps.

#### African animal trypanosomosis data

As for tsetse, data on AAT were mainly collected during baseline surveys and monitoring activities. During investigations, livestock from a given area were assembled in one site, randomly selected for screening, and the buffy-coat technique (BCT) was used to detect trypanosome infections [50]. AAT field data sheets capture information on the name, geographic coordinates and administrative units of the survey site, date of the survey, name of the farmer, number of animals present and sampled, number of animals that tested positive for trypanosomosis (by trypanosome species), packed cell volume (PCV) and age of the animal. An AAT data sheet normally includes information for 10 to 30 animals.

### Structure of the atlas

The KENTTEC atlas is composed of a data repository and a database. The database is constituted by two simple spreadsheets (Microsoft Excel), one for tsetse and one for AAT, each file including one single sheet. The detailed structure of the files, i.e. their column by column description, is provided in Additional file 1: Text S1.

The data repository includes all field data sheets used as input for the atlas. In the repository, spreadsheets are organized by type of data (tsetse or AAT) and by period of data collection (i.e. year). To complement field data sheets, especially in a few situations when some data had gone missing, narrative reports of field activities were included in the repository, and they were used to extract data for the atlas. Overall, the data repository includes 757 recording data sheets for AAT and 467 sheets for tsetse.

The tsetse component of the database includes information on the source used (i.e. the data recording sheet in the data repository), the geographical location of the trapping site, i.e. administrative units and geographic coordinates (latitude and longitude in decimal degrees), the type of trap and odour attractant, the period of survey, tsetse species (including number of flies caught, disaggregated by sex, and apparent density). The presence or absence of KENTTEC control activities against tsetse is also recorded, which allows to distinguish baseline from monitoring data.

The AAT component of the database captures information on the survey/monitoring sites (i.e. location name, administrative units and geographic coordinates), tested animals (species, breed, age and sex), diagnostic method, the survey period, sample size, and the results of the survey in terms of number and prevalence of trypanosomal infections (disaggregated by trypanosome species) and PCV. The presence or absence of KENTTEC control activities against tsetse is also recorded.

### Development of the atlas

From the start of the PATTEC project in Kenya in 2006, the data recording sheets compiled in the field were routinely transmitted in hard copy to HQ in Nairobi. Occasionally, a few of them were not submitted, but a narrative report was made available to HQ instead. Most data sheets were converted in digital format at HQ, while a few attempts were made to digitize them in the field stations.

KENTTEC’s initiative to develop the atlas was launched in 2016. A focal point for data management and mapping at the national level was identified and appointed, and efforts were renewed to collate all existing data from field offices. Hard copy data were systematically digitized, cross-checked and assembled in the data repository. Thorough data clean-up, verification, harmonization and completion was also undertaken, to ensure consistency and completeness. Free and Open Source Geographic Information System (GIS) software (Quantum GIS) was used, especially to verify the accuracy of the GPS coordinates recorded in the field. Standardization of the format of geographical coordinates was carried out (i.e. all coordinates were converted into latitude and longitude on WGS84 datum, decimal degrees). The format of survey dates and the values of various attributes (tsetse species, trap type, geographical location, etc.) were also standardized. Importantly, the names of the source input files were recorded and harmonized, thus enabling cross-verifications to be carried out. Data completion was mainly needed in those instances when some information had not been captured in the original recording sheets, e.g. AAT prevalence, when only the number of infections were recorded, tsetse apparent densities (number of flies/trap/day), when only the number of catches were reported, and duration of the survey, when only the start and end date of the survey were recorded. Other items included during data completion relate to the random selection of animals in AAT surveys, and the presence of KENTTEC interventions against tsetse and AAT (or lack thereof). The latter was normally inferred from narrative reports of field officers. In the AAT input datasets, a number of gaps had to be filled for geographical coordinates. In most instances, the recorded village name enabled coordinates to be extracted from alternate sources (e.g. gazetteers and Google Earth). Finally, AAT data collected in the field at the animal level were aggregated at the herd/site level for inclusion in the database.

## Results

### Tsetse distribution

A total of 6254 tsetse trapping events were recorded in the atlas (1924 in the framework of baseline surveys and 4330 from monitoring activities), in approximately 5000 different trapping locations. The overall trapping intensity was 15,284 trap days. In these entomological surveys, a total of 71,662 tsetse flies were caught, with an average apparent density of 7 flies/trap/day in baseline surveys and 3.5 flies/trap/day in monitoring surveys. Six species of *Glossina* were caught, namely *G. pallidipes* (*n *= 62,424 flies, i.e. 87% of the total catches), *G. brevipalpis* (*n* = 5597, 8%), *G. f. fuscipes* (*n* = 2835, 4%), *G. longipennis* (*n* = 468, < 1%), *G. austeni* (*n* = 168, < 1%) and *G. swynnertoni* (*n* = 123, < 1%).

**Fig. 1:**
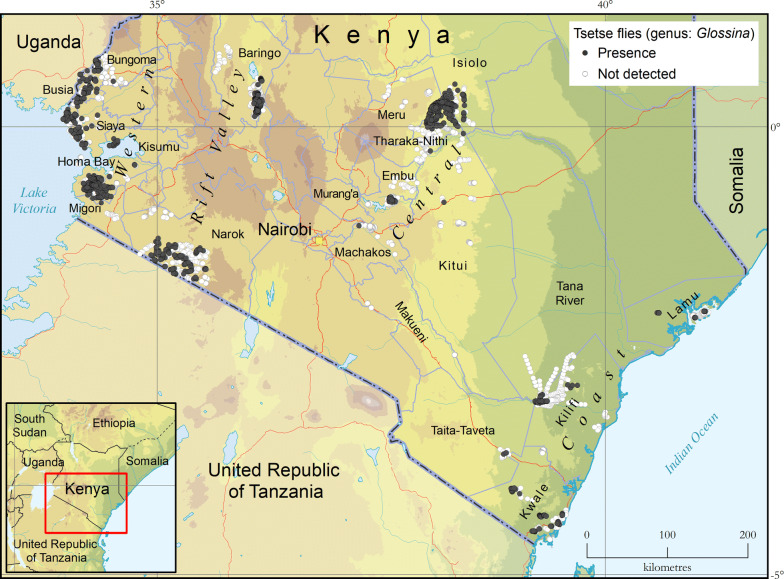
Presence (black circles) and absence (surveyed but not detected, white circles) of tsetse flies (genus *Glossina*) in Kenya. Data collection period: 2006–2019 Source: Kenya Tsetse and Trypanosomosis Eradication Council (KENTTEC)

As shown in Fig. 1, tsetse fly presence was confirmed in all four study regions (i.e. Western, Rift Valley, Central and Coast) and in 18 out of 21 study counties (i.e. in all counties except for Makueni, Migori and Tana River, where the geographical coverage of the surveys was limited).

**Fig. 2:**
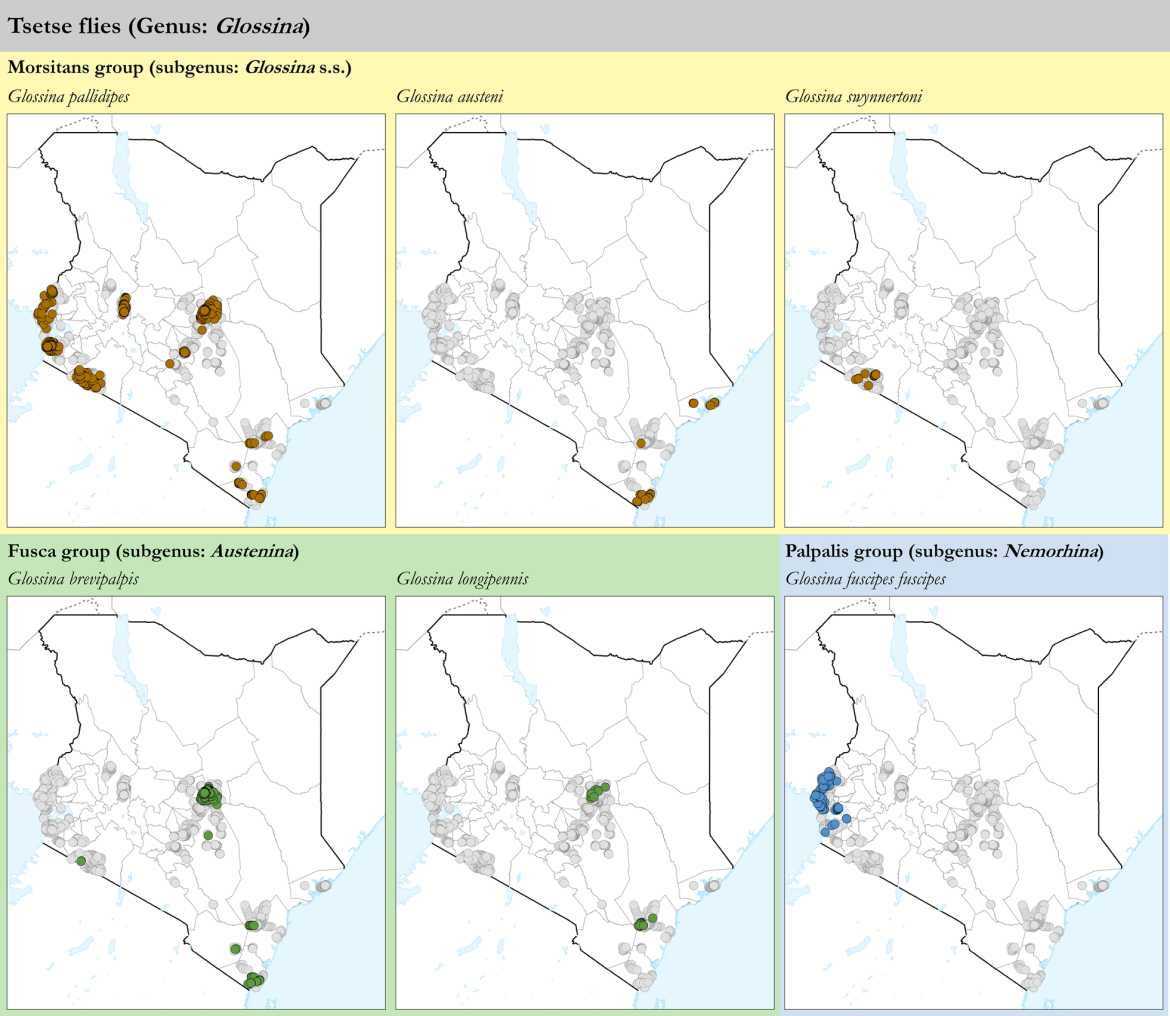
Presence (coloured circles) and absence (surveyed but not detected, grey circles) of tsetse fly species in Kenya. Data collection period: 2006–2019 Source: Kenya Tsetse and Trypanosomosis Eradication Council (KENTTEC)

Looking at the different species (Fig. 2), *G. pallidipes* (morsitans group) is by far the one with the broadest geographical distribution, its presence having been confirmed in all study regions and in 14 out of 21 study counties. As for the other savannah species, *G. austeni* was only found in the Coast region and *G. swynnertoni* only in the southern part of the Rift Valley region that corresponds to the Maasai Mara National Reserve (Narok county). As to the fusca group, the presence of two species was confirmed. *Glossina brevipalpis* was detected in all but the Western region, and *G. longipennis* was found in the Central and Coast regions*. Glossina fuscipes fuscipes*, the only tsetse species of the palpalis group present in Kenya, was only found in the Western region.

### African animal trypanosomosis distribution

For the AAT component of the atlas, a total of 898 surveys were included in the database, corresponding to 537 different locations/sites. Out of these surveys, 286 were baseline (i.e. pre-intervention) and 612 were monitoring (i.e. during or post-intervention). A total of 49,785 domestic animals were screened [i.e. 48,806 bovines (98.03%), 809 caprines (1.62%), 84 ovines (< 1%), 34 equines, 31 canines and 21 porcines]. Overall, 914 animals (1.84%) were found positive for trypanosome infection. The average baseline prevalence was 3.2%, while the average prevalence during monitoring was 1.3%.

**Fig. 3:**
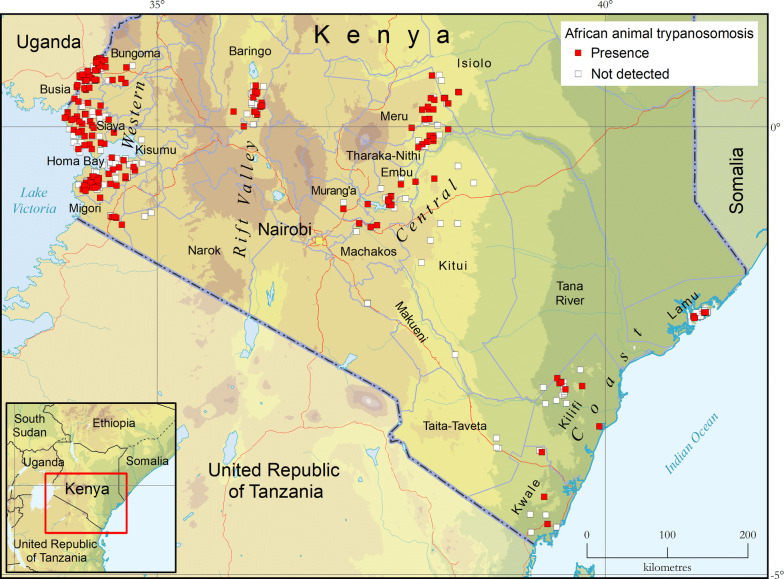
Presence (red squares) and absence (surveyed but not detected, white squares) of trypanosome infection in various domestic animal species (cattle, goats, sheep, donkeys, dogs and pigs) as determined with the buffy-coat technique (BCT). Study period: 2006–2019 Source: Kenya Tsetse and Trypanosomosis Eradication Council (KENTTEC)

**Fig. 4:**
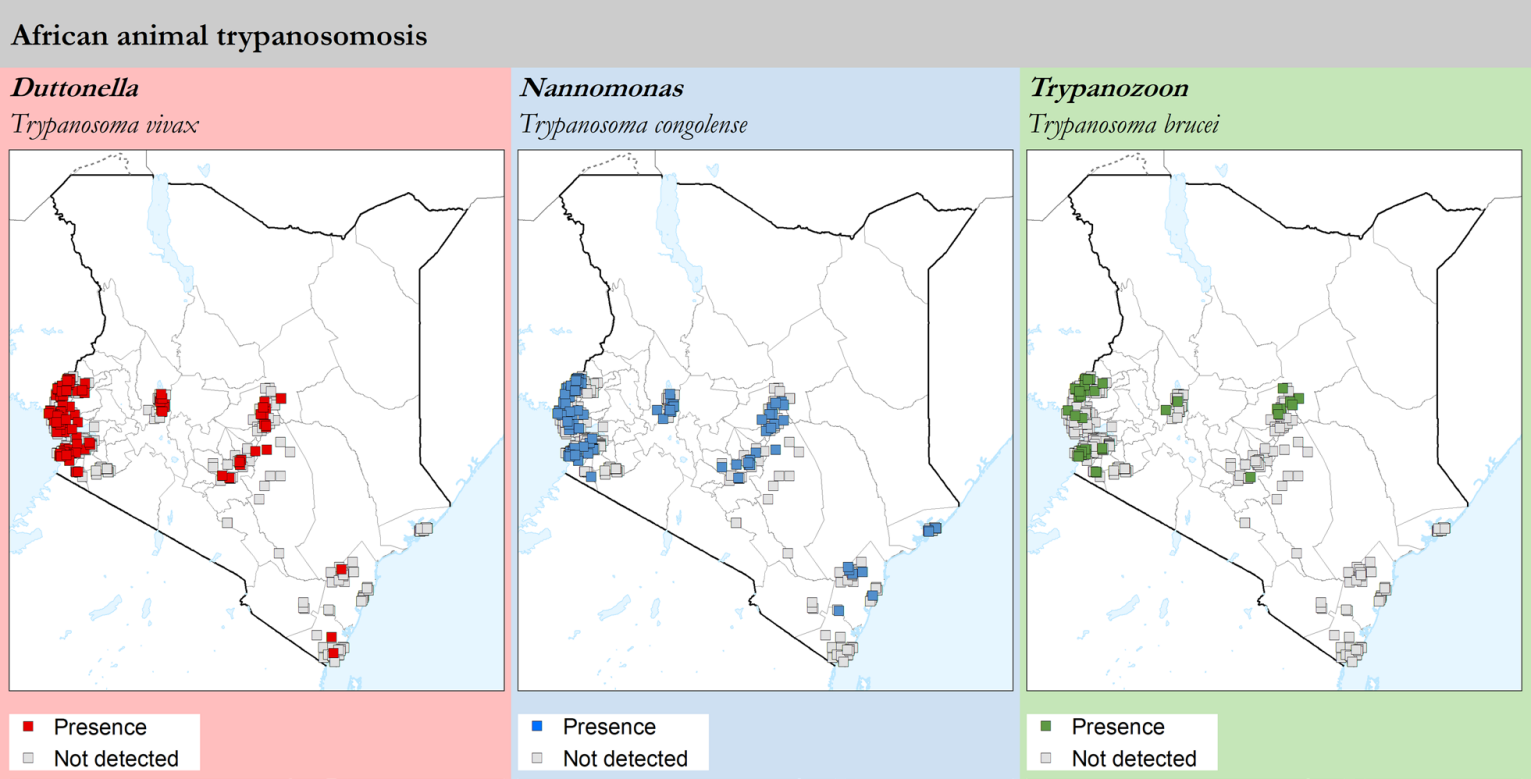
Presence (coloured squares) and absence (surveyed but not detected, grey squares) of *T. vivax*, *T. congolense* and *T. brucei* in various domestic animal species (cattle, goats, sheep, donkeys, dogs and pigs) as determined with the buffy-coat technique (BCT). Study period: 2006–2019 Source: Kenya Tsetse and Trypanosomosis Eradication Council (KENTTEC)

AAT surveys were carried out alongside entomological investigations in the four study regions, and results in terms of presence or absence of detection are summarized in Fig. 3. Disaggregated data for *T. vivax*, *T. congolense* and *T. brucei* are shown in Fig. 4.

Animal trypanosomosis was detected in all investigated regions. *Trypanosoma vivax* (444 infections, 0.89% prevalence) and *T. congolense* (390 infections, 0.78% prevalence) are the two trypanosome species with the broadest geographical range, having been found in all regions. *Trypanosoma brucei* was detected at much smaller rates (i.e. 96 infections, 0.19% prevalence), and was not detected in the Coast region. Only 16 *Trypanosoma* spp. co-infections were detected.

### Completeness of the database

For the tsetse component of the database, completeness is very high, as information for most of the required fields could be identified either in the recording sheets or in the complementary reports. In particular, a very high level of completeness was achieved for geographical coordinates (99.8%), dates of tsetse trapping (99.9%), captured tsetse species, number of flies caught and the related apparent densities (100%), as well as tsetse interventions (100%). ‘Trap type’ is the least complete information item, because information on the type of trap was not available for 64% of the tsetse records.

In the AAT component, database completeness is also very high, if somewhat lower than the tsetse component [geographical coordinates (95% complete), dates of screening (97%), trypanosome species (100%), number of infections and the related disease prevalence (99%)]. Major gaps affect the fields ‘husbandry system’ (not available for any survey), and the ‘breed’ (not available for 80% of the surveys). These major gaps owe to the fact that this type of information is not included in the field recording sheets.

## Discussion

The present paper describes the atlas of tsetse and AAT in Kenya, an initiative implemented by KENTTEC and other national and international stakeholders. Preliminary results presented here are based on data collected by KENTTEC over a 14 year period (2006–2019). The atlas is the largest database of tsetse distribution in Kenya developed in over 20 years, and it is also the first attempt to collate and map AAT data at the national level. Importantly, the maps presented here are underpinned by a dynamic information system, which will enable future upgrades and updates to be made.

At the present stage of development, the most notable geographical gap in the atlas is related to the northern and north-eastern parts of the country (e.g. West Pokot, Turkana, Samburu, Marsabit and Garissa counties). Even though large swaths of territory in these counties have been historically free of tsetse [22], and although there is little, if any, recent published evidence of tsetse infestation [19], pocket populations are expected to be still present. Furthermore, albeit tsetse-free, many of these areas could nonetheless be affected by AAT because of the combined effects of animal movement and mechanical transmission [9]. Smaller but still significant geographical gaps also affect the southern parts of the country (e.g. Kajiado county, and large areas in Makueni, Kitui and Tana River counties), where some areas such as the Nguruman escarpment and the Kibwezi forest are known to be infested by tsetse [19, 23].

**Fig. 5:**
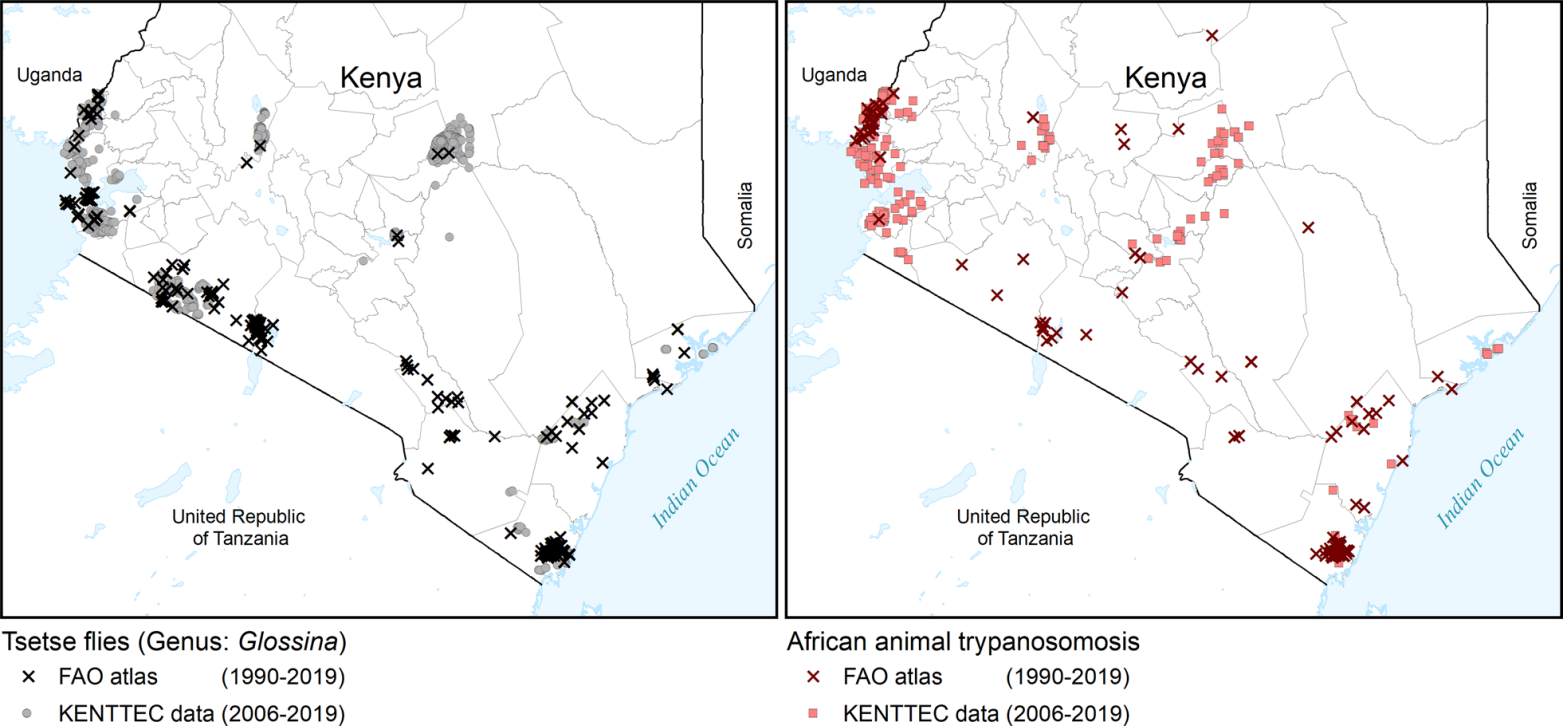
Data from the FAO continental atlas of tsetse and ATT (period 1990–2019) overlaid on the KENTTEC data (2006–2019). FAO data are extracted from systematic reviews of scientific publications [19, 32]

A few of the geographical gaps in the atlas could be filled by already published research data. For example, Fig. 5 shows data from the FAO continental atlas overlaid on the KENTTEC data. FAO data, as extracted from a systematic review of 30 years of scientific publications [19, 32], have been provided to KENTTEC and they can already be used to complement the national atlas. However, for a proper inclusion of research data in the national atlas, the raw data underpinning publications should be collated by KENTTEC, and the full engagement of the partner institutions will be crucial.

Beyond the geographical gaps, other limitations affect the atlas. With regard to AAT, all data were collected with low-sensitivity parasitological methods, notably, BCT. Also, 98% of the tested animals are cattle, despite the fact that the number of small ruminants in the country is more than double that of cattle. The diagnostic of choice and the focus on cattle are also likely to have contributed to the lack of detection of *T. simiae*, a species of trypanosome that is known to circulate in Kenya [51, 52].

With regard to tsetse, one of the weaknesses of the atlas is that no mobile device was used in the surveys, even though, for certain species of tsetse, moving targets may be more attractive than stationary baits [49]. Also, no specimens of *G. morsitans submorsitans* (savannah group) and *G. fuscipleuris* (forest group) were captured. With regard to these two species, a recent study confirmed the presence of *G. fuscipleuris* [20]; by contrast, the small zone in the North-West (Turkana county) where *G. morsitans submorsitans* was historically reported [23] was not investigated, so its continued presence in Kenya cannot be ruled out.

**Fig. 6:**
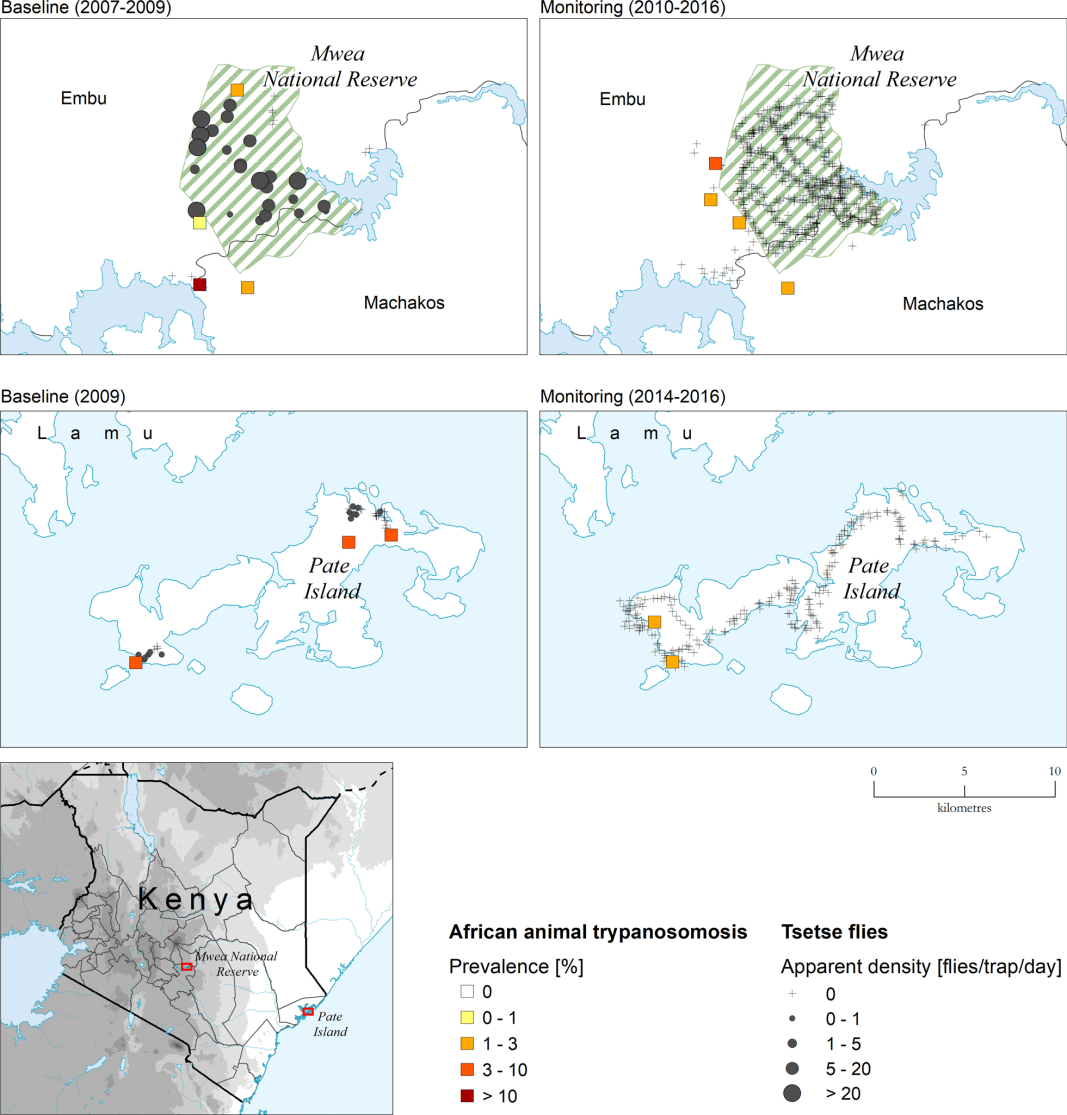
Comparison of baseline and monitoring data in two PATTEC Kenya/KENTTEC interventions areas: Mwea National Reserve (central Kenya) and Pate island (costal Kenya, Lamu Archipelago). Baseline data: 2007–2009 (Mwea National Reserve) and 2009 (Pate Island). Monitoring data: 2010–2016 (Mwea National Reserve) and 2014–2019 (Pate Island)

One of the main assets of national atlases of tsetse and AAT is the ease with which data for different time periods can be compared. In the case of the KENTTEC dataset, comparison between baseline (i.e. pre-intervention) and monitoring (i.e. during or post-invention) is the most interesting. A comprehensive evaluation of the impact of PATTEC Kenya/KENTTEC interventions over the entire study period is beyond the scope of the present paper. However, Fig. 6 exemplifies how the atlas can assist such an impact assessment. The example focuses on two of the areas where tsetse control was particularly successful, i.e. Mwea National Reserve (Central region) and Pate island (Lamu county, Coast region). In these two areas, tsetse flies were reduced below detectable levels, and they may have been eliminated, while AAT appears to have been reduced but not eliminated.

In several other intervention areas (not discussed here in detail), the impact of interventions was less dramatic, but various levels of reduction were observed in tsetse densities and/or AAT prevalence.

At the present stage of development, the atlas in Kenya cannot be considered complete, because only KENTTEC data have been included so far. However, mechanisms are being put in place and agreements are being formalized to ensure the full engagement of KENTTEC’s partner institutions. These are, among others, the Biotechnology Research Institute and the Veterinary Science Research Institute of the Kenya Agricultural & Livestock Research Organization (KALRO), the Directorate of Veterinary Services, the Ministry of Health, the International Centre of Insect Physiology and Ecology (ICIPE), the International Livestock Research Institute (ILRI), the Kenya Wildlife Services and other learning and academic institutions (e.g. the Kenyatta University and the University of Nairobi). With KENTTEC’s technical assistance, these partner institutions are in the process of assembling and harmonizing their data with a view towards developing a comprehensive information system/atlas on tsetse and AAT in Kenya.

Data from partner institutions will allow to fill some of the geographical gaps that presently affect the atlas. However, it is known that in certain zones, especially in the North, no survey has been carried out in decades. Most of the areas in northern Kenya are characterized by pastoral production systems, where livelihoods rely heavily on livestock [53]. New surveys are therefore needed in these zones to clarify the epidemiological situation, and to inform the development of appropriate strategies for trypanosomosis control. Geospatial modelling and remote-sensing could help target future surveys by mapping habitat suitability for tsetse [54]. Geospatial tools could also be combined with genetic data to estimate the degree of isolation of the different tsetse populations [55, 56], a key factor in guiding the choice between reduction or elimination strategies [36].

## Conclusions

Proper management of epidemiological data is crucial to plan and monitor interventions against tsetse and trypanosomosis. However, most endemic countries lack effective information systems to manage data at the national level. The work presented here highlights the efforts made in Kenya to tackle this shortfall. Future efforts to facilitate the regular and speedy update of the atlas should include capacity building for field data collectors. Enhancing the format of data recording sheets is also needed, so that all information items required for the atlas can be collected in a comprehensive and harmonized manner in the field. Looking beyond the entomological and epidemiological data assembled in the atlas, there is a need to improve the management of control data. These should include information on both direct interventions against AAT (i.e. the administration of curative or prophylactic trypanocidal drugs), and interventions against tsetse [e.g. ITT, ITC, ground or aerial spraying [57], the sterile insect technique (SIT) [58], etc.)]. The joint assessment of the control data and of the epidemiological situation as captured in the atlas will allow to establish the PCP stage for each area of the country (i.e. where each area stands in the pathway of progressive control of AAT) [36]. This approach is being promoted by KENTTEC in its strategic planning exercises. In particular, a comprehensive, county-level PCP staging and mapping is envisaged, which will inform the selection of the most appropriate control strategy for each area.

## Supplementary information

**Additional file 1: Text S1.** Structure of the database on tsetse and African animal trypanosomosis in Kenya.

## Data Availability

Data supporting the conclusions of this article are included within the article and its Additional file 1. The bulk of the data on the tsetse and AAT occurrence in Kenya is the property of the Government of Kenya, (Kenya Tsetse and Trypanosomosis Eradication Council), and data can be requested to: Chief Executive Officer, Kenya Tsetse and Trypanosomosis Eradication Council, PO Box: 66290-00808, Nairobi, Kenya, Phone: +254 20 2513131, E-mail: info@kenttec.go.ke

## Abbreviations

AAT: African animal trypanosomosis
AfDB: African Development Bank
BCT: buffy-coat technique
FAO: Food and Agriculture Organization of the United Nations
GPS: Global Positioning System
HAT: human African trypanosomosis
HQ: Head Quarters
IAEA: International Atomic Energy Agency
ICIPE: International Centre of Insect Physiology and Ecology
ILRI: International Livestock Research Institute
ITC: insecticide-treated cattle
ITT: insecticide-treated targets
KALRO: Kenya Agricultural & Livestock Research Organization
KENTTEC: Kenya Tsetse and Trypanosomosis Eradication Council
NEPAD: New Partnership for Africaʼs Development
PAAT: Programme Against African Trypanosomosis
PATTEC: Pan-African Tsetse and Trypanosomosis Eradication Campaign
PCP: progressive control pathway
PCV: packed cell volume
UTM: universal transverse mercator
WGS84: World Geodetic System 1984
WHO: World Health Organization
