# Supplementary material for: Developing a national atlas to support the progressive control of tsetse-transmitted animal trypanosomosis in Kenya
Source: Parasit Vectors. 2020 Jun 5;13:286. doi: 10.1186/s13071-020-04156-5 (PMC7275614; doi:10.1186/s13071-020-04156-5)
Supplement: Supplementary file 1 — Additional file 1: Text S1. Structure of the database on tsetse and African animal trypanosomosis in Kenya. [file 13071_2020_4156_MOESM1_ESM.docx]

# Additional file 1: Text S1

# Structure of the atlas of tsetse and AAT in Kenya

**Tsetse flies table**

1. SOURCE_ID. A unique numeric identifier of the data source examined.
2. SOURCE_NAME. Name of the data source examined.
3. TRAPPING_SITE_ID. A unique numeric identifier of the trapping site.
4. TRAP_NAME. Trap name in the original data source (e.g. the original field data recording sheet).
5. LONG. Longitude of the trapping site in decimal degrees (Datum: WGS84)
6. LAT. Latitude of the trapping site in decimal degrees (Datum: WGS84)
7. REGION. Name of the PATTEC/KENTTEC intervention region where the trapping was carried out.
8. ADMIN1_County. Name of the first subnational administrative unit (County) where the site is located.
9. ADMIN2_Subcounty. Name of the second subnational administrative unit (Subcounty) where the site is located.
10. ADMIN3_Location. Name of the third subnational administrative unit (Location) where the site is located.
11. ADMIN4_Sublocation. Name of the fourth subnational administrative unit (Sublocation) where the site is located.
12. LOCALITY_NAME. Name of the locality where the trapping was conducted. As a rule, the name of the closest village is recorded.
13. GEO_SOURCE. Source of geographic coordinates of the trapping site (usually the GPS coordinates recorded in the original data source).
14. TRAPPING_EVENT_ID. Unique numeric identifier of the trapping event (a trapping event is a single trap deployed in a given site at a given period of time) .
15. TSETSE_ID. A unique numeric identifier of each record in the tsetse table. If more than one tsetse species/subspecies is detected in given trapping event (i.e. in a given TRAPPING_EVENT_ID), each tsetse species/subspecies will be assigned a different TSETSE_ID.
16. DAY_ST. Starting day of the trapping event.
17. MONTH_ST. Starting month of the trapping event.
18. YEAR_ST. Starting year of the trapping event.
19. DAY_EN. Ending day of the trapping event.
20. MONTH_EN Ending month of the trapping event.
21. YEAR_EN. Ending year of the trapping event.
22. START_DATE. Starting date (dd/mm/yyyy) of the trapping event.
23. END_DATE. Ending date (dd/mm/yyyy) of the trapping event.
24. TRAP_TYPE. Type of trap used.
25. TRAP_ATTRACTANT. Odour attractant used in the trap.
26. TRAP_TIME. Duration of trapping (in hours) for the given trapping event (usually, either 48 or 96 hours).
27. SPECIES. Name of the species or subspecies of tsetse detected in a trapping event. When data refer to tsetse flies as a Genus (i.e. not to individual species) “Genus: *Glossina”* is used.
28. MALE_NT. Non teneral male tsetse detected.
29. MALE_T. Teneral male tsetse detected.
30. FEMALE_NT. Non teneral female tsetse detected.
31. FEMALE_T. Teneral female tsetse detected.
32. FLIES_Number. Total number of tsetse detected.
33. FLIES_AD. Tsetse apparent density (flies/trap/day).
34. FLIES_Absence_Presence. Tsetse absence/presence (Presence: Yes, Absence: No).
35. OTHER_SPECIES. Presence of information on other species/subspecies in the same trapping event. ‘Yes’: other species/subspecies were detected in the same trapping event. ‘No’: no other species/subspecies was detected in the same trapping event
36. Baseline_monitoring. It indicates whether data were collected before the start of tsetse control interventions by PATTEC/KENTTEC (i.e. baseline) or during/after interventions (i.e. monitoring).

**African animal trypanosomosis table**

1. SOURCE_ID. A unique numeric identifier of the data source examined.
2. SOURCE_NAME. Name of the data source examined.
3. SURVEY_SITE_ID. A unique numeric identifier of the site where the survey was carried out.
4. LAT. Latitude of the survey site in decimal degrees (Datum: WGS84).
5. LONG. Longitude of the survey site in decimal degrees (Datum: WGS84).
6. REGION. Name of the PATTEC/KENTTEC intervention region where the survey was carried out.
7. ADMIN1_County. Name of the first subnational administrative unit (County) where the site is located.
8. ADMIN2_SubCounty. Name of the second subnational administrative unit (Sub-county) where the site is located.
9. ADMIN3_Location. Name of the third subnational administrative unit (Location) where the site is located.
10. ADMIN4_Sublocation. Name of the fourth subnational administrative unit (Sublocation) where the site is located.
11. LOCALITY_NAME. Name of the locality where the survey was conducted. As a rule, the name of the closest village is recorded.
12. GEO_SOURCE. Source of geographic coordinates of the survey (usually the GPS coordinates recorded in the original data source, e.g. field data recording sheet, or gazetteers, Google Earth, etc.).
13. GEOREFERENCING_NOTES. It includes details on the approach used for geo-reference the survey site.
14. SURVEY_ID. A unique numeric identifier of the survey (a survey is a sample on animals in a given site at a given period of time).
15. DATE. Date of the survey.
16. MONTH_ST. Starting month of the survey.
17. YEAR_ST. Starting year of the survey.
18. MONTH_EN. Ending month of the survey.
19. YEAR_EN. Ending year of the survey.
20. SAMPLE_SIZE: number of animals sampled.
21. SPECIES_AN: species of animal sampled.
22. BREED_AN: animal breed.
23. AGE_AN: age of animals.
24. SEX_AN: sex of animals.
25. HUSB_AN: Animal husbandry system.
26. Infections with individual trypanosome species
    - Tv: Number of animals positive to the test for *Trypanosoma vivax*
    - Tc: Number of animals positive to the test for *T. congolense*
    - Tb: Number of animals positive to the test for *T. brucei*
    - Tsi: Number of animals positive to the test for *T. simiae*
27. T: Number of animals positive to the test for any of the four species of trypanosomes under study (*T. vivax*, *T. congolense*, *T. brucei* and *T. simiae*).
    - Note that animals diagnosed with a mixed infection (infections with more than one species of trypanosomes) are included in the counts for the respective infections with individual species.
28. TPR [%]: Total AAT Prevalence (in percentage), including infections with any of the four species of trypanosomes under study (*T. vivax*, *T. congolense*, *T. brucei* and *T. simiae*).
    - Separate values for the prevalence of individual species of trypanosomes are also included in the database, but they are not listed in this document.
29. AAT_PRESENCE: ‘Yes’ if African animal trypanosomosis was detected, “No” otherwise.
30. DIAGNOSTIC: Diagnostic method used for trypanosome identification.
31. PCV: Average Packed-Cell-Volume for all animals tested in the survey.
32. PCV_POSITIVE: Average Packed-Cell-Volume for the animals that were positive to the test for trypanosomosis.
33. PCV_NEGATIVE: Average Packed-Cell-Volume for the animals negative to the test for trypanosomosis.
34. Baseline/Monitoring: it indicates whether data were collected before the start of tsetse control interventions by PATTEC/KENTTEC (i.e. baseline) or during/after interventions (i.e. monitoring).
35. SAMPLING_STRATEGY. It describes whether a random sampling of animals was used (i.e. to assess the general epidemiological situation in an area), or a purposeful one (e.g. where specific animals such as clinically suspicious ones are tested).
